# Domain analysis reveals striking functional differences between the regulatory subunits of phosphatidylinositol 3-kinase (PI3K), p85α and p85β

**DOI:** 10.18632/oncotarget.19866

**Published:** 2017-08-03

**Authors:** Yoshihiro Ito, Peter K. Vogt, Jonathan R. Hart

**Affiliations:** ^1^ Department of Molecular Medicine, The Scripps Research Institute, La Jolla, San Diego, CA, USA

**Keywords:** SH2 domain, SH3 domain, RhoGAP domain, iSH2 domain, domain exchange

## Abstract

Our understanding of isoform-specific activities of phosphatidylinositol 3-kinase (PI3K) is still rudimentary, and yet, deep knowledge of these non-redundant functions in the PI3K family is essential for effective and safe control of PI3K in disease. The two major isoforms of the regulatory subunits of PI3K are p85α and p85β, encoded by the genes PIK3R1 and PIK3R2, respectively. These isoforms show distinct functional differences that affect and control cellular PI3K activity and signaling [1–4]. In this study, we have further explored the differences between p85α and p85β by genetic truncations and substitutions. We have discovered unexpected activities of the mutant proteins that reflect regulatory functions of distinct p85 domains. These results can be summarized as follows: Deletion of the SH3 domain increases oncogenic and PI3K signaling activity. Deletion of the combined SH3-RhoGAP domains abolishes these activities. In p85β, deletion of the cSH2 domain reduces oncogenic and signaling activities. In p85α, such a deletion has an activating effect. The deletions of the combined cSH2 and iSH2 domains and also the deletion of the cSH2, iSH2 and nSH2 domains yield results that go in the same direction, generally activating in p85α and reducing activity in p85β. The contrasting functions of the cSH2 domains are verified by domain exchanges with the cSH2 domain of p85β exerting an activating effect and the cSH2 domain of p85α an inactivating effect, even in the heterologous isoform. In the cell systems studied, protein stability was not correlated with oncogenic and signaling activity. These observations significantly expand our knowledge of the isoform-specific activities of p85α and p85β and of the functional significance of specific domains for regulating the catalytic subunits of class IA PI3K.

## INTRODUCTION

We recently reported an oncogenic activity of wild type p85β in cultures of avian embryonic fibroblasts [2]. This activity manifests itself in cellular transformation that alters cell morphology and increases the growth potential of the cells. It is also correlated with enhanced PI3K signaling as documented by elevated phosphorylation of AKT at S473. Wild type p85α lacks such oncogenic activity. The causes for this distinct difference in the biological and biochemical properties of these two p85 isoforms are not known. The p85α and p85β proteins are encoded by two different genes, PIK3R1 and PIK3R2, but show extensive protein sequence homology. Both consist of five structurally and functionally defined domains. They are, starting at the N-terminus: an SH3 domain, a RhoGAP domain (also referred to as breakpoint-cluster region homology (BH) domain that is flanked by two proline-rich regions), an N-terminal SH2 domain (nSH2), an inter-SH2 region (iSH2), and a C-terminal SH2 (cSH2) domain [1, 3]. The p85 proteins can interact with a multitude of proteins involved in cellular signaling [1, 3]. The most important interactions are an inhibitory binding of the nSH2 domain to the helical domain of the catalytic subunit p110 of PI3K and, for class IA PI3Ks, activating interactions of the SH2 domains with upstream signaling proteins containing targeted phosphotyrosine residues [5–9].

Here we have investigated the biological and biochemical activities of p85 domains comparing the α and β isoforms. The results provide new insights into the functions of the p85 domains. These include an inhibitory action of the SH3 domain and essential roles of the RhoGAP and cSH2 domains in oncogenic signaling. These studies also reveal functional differences between the p85α and p85β cSH2 domains; the hinge domain located between iSH2 and cSH2 contributes to these differences.

## RESULTS

### The SH3 domain of p85 inhibits PI3K signaling

**Figure 1:**
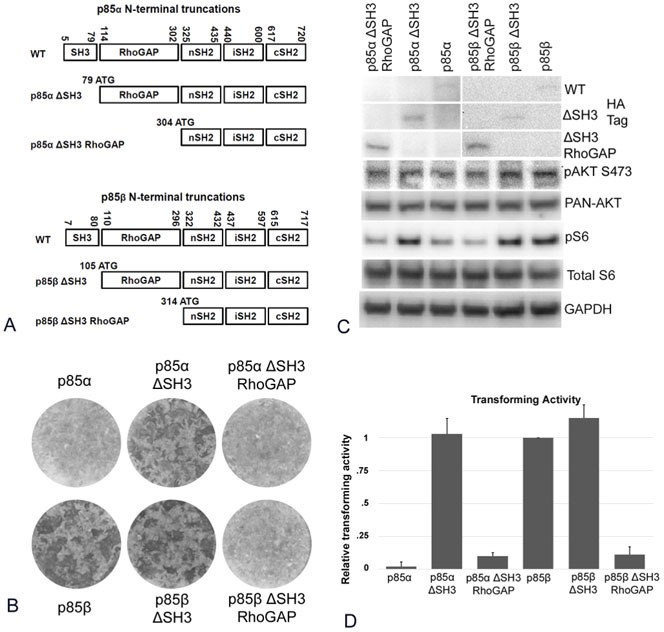
N-terminal truncations of p85 **A.** Schematic representation of the p85 N-terminal truncation mutants. **B.** Representative focus assays of p85 N-terminal truncations. **C.** Activation of PI3K signaling in cells expressing N-terminally truncated p85 as reflected by Western blots of Akt phosphorylated at S437 and S6 at Ser235/236. The data in Figure 1C are from a representative experiment that was repeated three times with consistent results (for details, see the Materials and Methods section). **D.** Efficiencies of cellular transforming activity of p85 N-terminal truncation mutants relative to the transforming activity of wild type p85β. CEF cells were transfected RCAS(A) constructs expressing the inserts p85β, p85β ΔSH3, p85β ΔSH3-RhoGAP, p85α, p85α ΔSH3, and p85α ΔSH3-RhoGAP. Transforming activity was standardized to 0.5 μg transfected DNA.

We cloned two N-terminal truncations of both the human PIK3R1 and PIK3R2 genes; in one, the SH3 domain was deleted, in the other, both SH3 and RhoGAP domains were deleted (Figure 1A). These constructs were expressed with the RCAS vector in chicken embryo fibroblasts (CEF). Expression of the SH3 domain truncation of both p85α (p85α ΔSH3) and p85β (p85β ΔSH3) increased the formation of oncogenically transformed cell foci as compared with the unmodified constructs (Figure 1B and 1D). However, the truncation of both SH3 and RhoGAP domains in both p85 isoforms (p85 ΔSH3-RhoGAP) resulted in a lack of oncogenic activity (Figure 1B and 1D). We also determined the level of PI3K signaling in p85 ΔSH3, wild type p85 and the p85 ΔSH3-RhoGAP expressing CEF for both isoforms. Signaling was determined by Western blotting using the phosphorylation of AKT at S473 and of S6 at Ser235/236 as markers. Compared to cells transfected with wild type p85, the SH3 truncations showed increased phosphorylation of AKT and of S6. No such increase was detected with the ΔSH3-RhoGAP deletions (Figure 1C). These data show that a deletion of the SH3 domain enhances both signaling and oncogenic activity of the p85 isoforms. The SH3 domain contains a proline-rich motif that in p85α has been reported to be a necessary mediator for efficient binding to PTEN. The interaction between PTEN and p85α protects PTEN from proteolytic degradation [10, 11] and the SH3 deletion would prevent that protection and result in increased PI3K activity. Our data suggest that the SH3 domain in both p85 isoforms exerts an inhibitory effect on PI3K signaling. In the case of p85α, this could reflect the known interaction with PTEN; in the case of p85β, the mechanism of SH3-mediated inhibition remains to be identified.

### The cSH2 domains of p85α and of p85β show opposite effects in the regulation of PI3K oncogenic signaling

**Figure 2:**
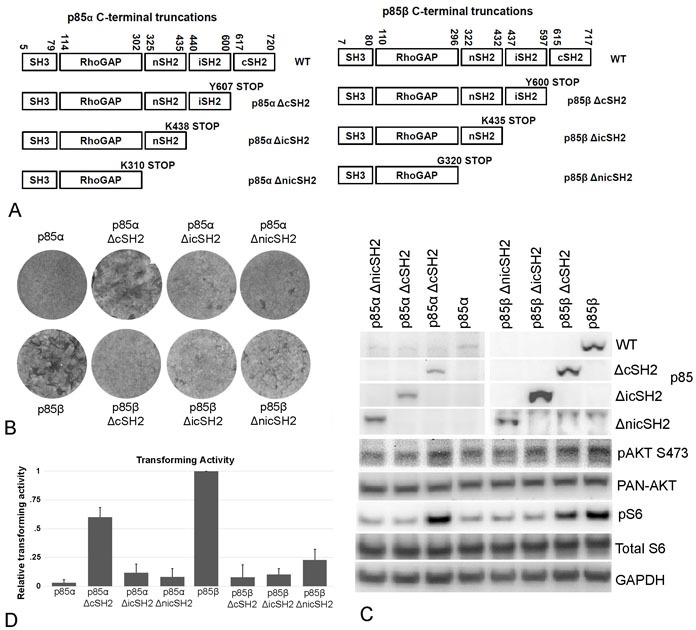
C-terminal truncations of p85 **A.** Schematic representation of the p85 C-terminal truncation mutants. **B.** Representative focus assays of p85 C-terminal truncations. **C.** PI3K signaling in cells expressing C-terminally truncated p85 as reflected by Western blots of Akt phosphorylated at S437 and S6 phosphorylated at Ser235/236. The data in Figure 2C are from a representative experiment that was repeated three times with consistent results (for details, see the Materials and Methods section). **D.** Efficiencies of cellular transforming activity of p85 C-terminal truncation mutants relative to the transforming activity of wild type p85β. CEF were transfected with RCAS(A) constructs expressing the inserts p85β, p85β ΔcSH2, p85β ΔicSH2, p85β ΔnicSH2, p85α, p85α ΔcSH2, p85α ΔicSH2, and p85α ΔnicSH2. Transforming activity was standardized to 0.5 μg transfected DNA.

We also analyzed C-terminal truncations of the p85 isoforms for their transforming and signaling activities. These constructs are depicted in Figure 2A and include deletions of the cSH2 domain (p85 ΔcSH2), the joint truncation of the iSH2 and cSH2 domains (p85 ΔicSH2) and deletion of the nSH2, iSH2 and cSH2 domains (p85 ΔnicSH2). It is known that a deletion of the cSH2 domain can enhance signaling activity of nSH2 mutants of p85α, and spontaneously occurring C-terminal truncations of p85α activate the oncogenic potential of p85α [2, 12] and of p85β [13]. Here we show that for p85α, truncation of the cSH2 domain by itself can activate focus-forming activity compared to the inactive wild type version of the protein (Figure 2B and 2D). This activation is correlated with enhanced phosphorylation of AKT S473 (Figure 2C). In contrast, the homologous truncation in p85β reduces focus-forming activity compared to wild type and attenuates AKT phosphorylation in CEF (Figure 2B - 2D). These observations suggest that the cSH2 domain of p85α acts as a negative regulating element for PI3K signaling, whereas in p85β, the cSH2 makes a positive contribution to the oncogenic and signaling activities of the wild type protein. Deleting both the iSH2 and cSH2 domains (ΔicSH2) reduced the activating effect of the cSH2 deletion in p85α and yielded a marginally active construct in the case of p85β (Figure 2B and 2D). The constructs expressing the triple truncation of the nSH2, iSH2 and cSH2 domains (ΔnicSH2) were not significantly different in activity from the aforementioned double truncations.

### Exchange of cSH2 and of iSH2 domains between p85α and p85β further documents functional differences between the two isoforms

**Figure 3:**
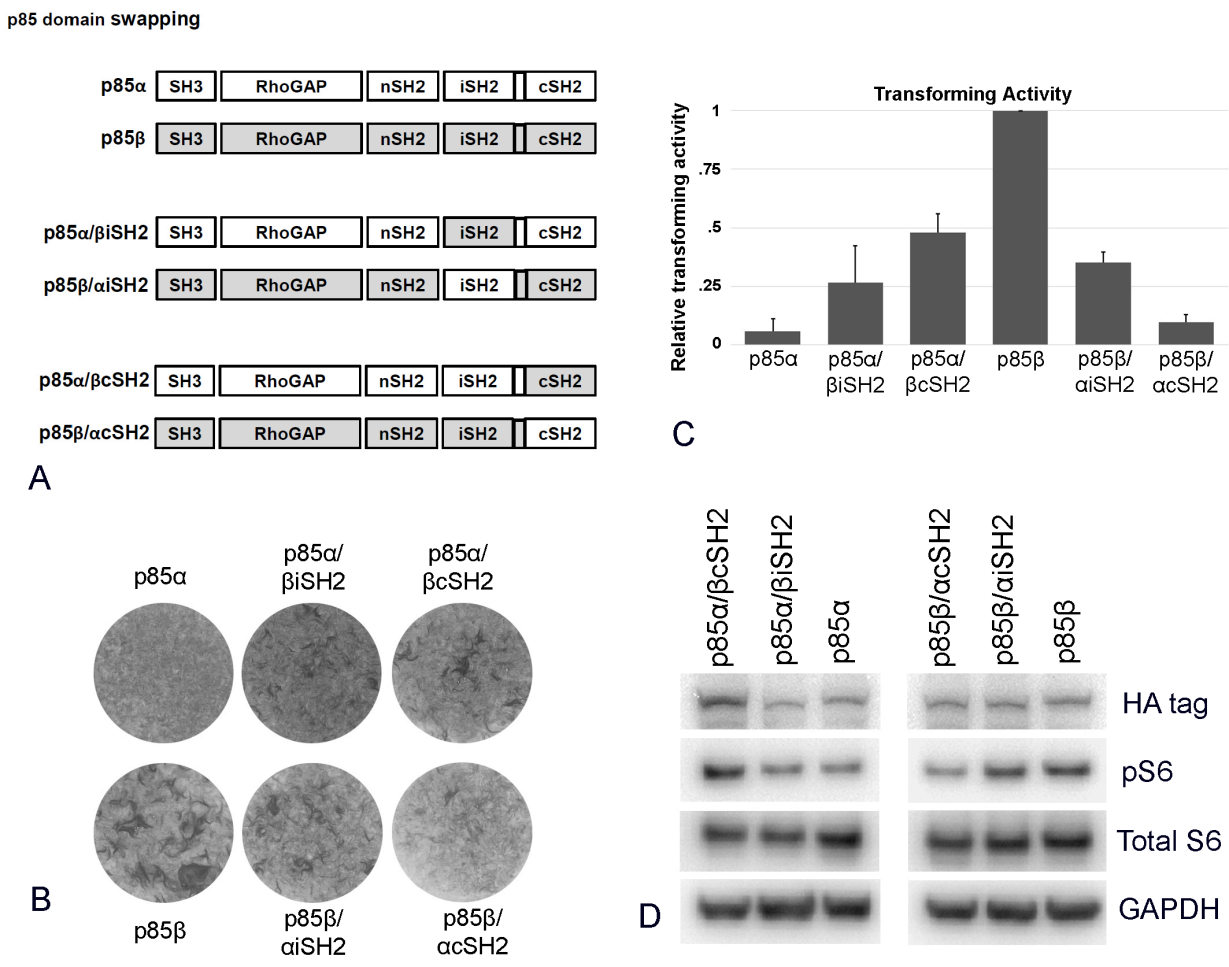
Exchanging cSH2 and iSH2 domains between p85α and p85β **A.** Schematic representation of the p85 cSH2 and iSH2 domain exchange mutants. **B.** Representative focus assays of p85 C-terminal domain exchange mutants. **C.** Efficiencies of cellular transforming activity of p85 domain exchange mutants relative to the transforming activity of wild type p85β. CEF were transfected with RCAS(A) constructs expressing the inserts p85β, p85α, p85β/αcSH2, p85β/αiSH2, p85α/βcSH2, and p85α/βiSH2. Transforming activity was standardized to 0.5 μg transfected DNA. **D.** PI3K signaling in cells expressing p85 domain exchange mutants as reflected by Western blots of S6 phosphorylated at Ser235/236. The data in Figure 3D are from a representative experiment that was repeated three times with consistent results (for details, see the Materials and Methods section).

We then exchanged the cSH2 and the iSH2 domains of p85α and p85β (Figure 3A). The cSH2 and iSH2 domains of p85β induce oncogenic activity in p85α; in contrast, wild type p85α has only very low focus-forming ability, and even this may not reflect wild type proper but spontaneously occurring activating C-terminal deletions [12] (Figure 3B and 3D). These domain exchanges did not appear to enhance signaling by p85α as reflected in AKT phosphorylation. However, the base value generated by wild type p85α probably reflects the occurrence of spontaneous activating mutations that routinely occur during retroviral expression of that protein. These make it impossible to rule out small yet significant differences between wild type and exchange mutant signaling [12] (Figure 3C). In contrast to these positive regulatory activities of the p85β domains, the p85α cSH2 and iSH2 domains have a negative effect on the oncogenic actions of p85β (Figure 3B - 3D). The cSH2 domain of p85α almost completely abolished the focus-forming activity of wild type p85β (Figure 3B and 3D). These observations expand the functional differences between p85α and p85β to the iSH2 domain. These differences probably reflect distinct interactions with p110 or with regulatory proteins.

### The different hinge regions between the iSH2 and cSH2 domains of p85α and p85β attenuate the activities of the cSH2 regions

**Figure 4:**
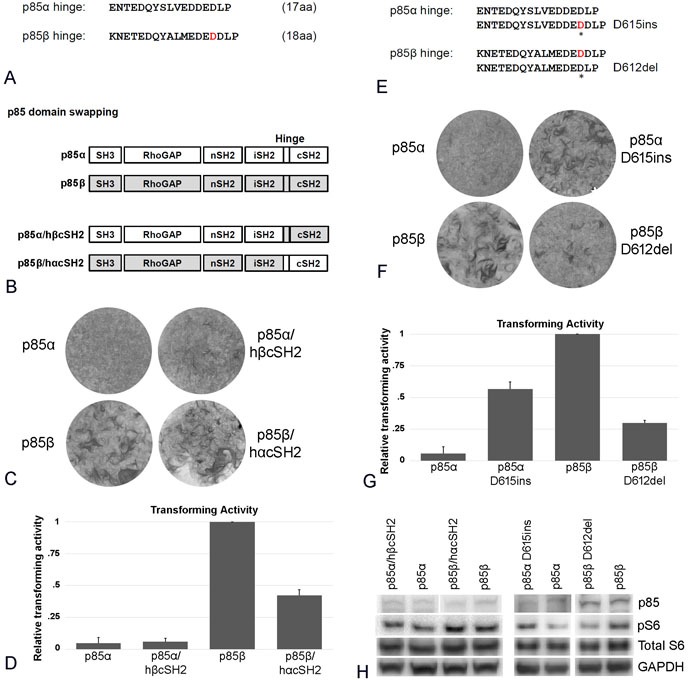
The effect of changing the p85 hinge region located between iSH2 and cSH2 **A.** Comparison of the amino acid sequences of the hinge regions of p85α and p85β. **B.** Schematic representation of the p85 hinge region constructs. **C.** Representative focus assays of p85α and p85β wild type and of the hinge region-cSH2 exchange constructs depicted in B. **D.** Efficiencies of cellular transforming activity of p85 hinge region constructs relative to the transforming activity of wild type p85β. CEF were transfected with RCAS(A) constructs expressing p85β, p85α, p85β/hαcSH2, p85α/hβcSH2). Transforming activity was standardized to 0.5 μg transfected DNA. **E.** Sequence of single amino acid insertions and deletion in the p85 hinge regions (p85α D615ins and p85β D612del). **F.** Representative focus assays of single amino acid insertions or deletions in the hinge region of p85. **G.** Efficiencies of cellular transforming activity of p85 single amino acid insertion or deletion mutant constructs relative to the transforming activity of wild type p85β. CEF were transfected with RCAS(A) constructs expressing p85α, p85β, p85αD615ins, and p85βD612del. Transforming activity was standardized to 0.5 μg transfected DNA. **H.** PI3K signaling in cells expressing p85 hinge region constructs and the single-amino acid insertion or deletion mutants as reflected by Western blots of S6 phosphorylated at Ser235/236. The data in Figure 4H are from a representative experiment that was repeated three times with consistent results (for details, see the Materials and Methods section).

The iSH2 and cSH2 domains of p85α and p85β are linked by a hinge region (Figure 4A). In p85α, the hinge region contains the residue that is the target of phosphorylation by p110 (S608). Phosphorylation of S608 results in downregulation of PI3K activity [14]. The hinge region of p85β lacks this phosphorylation site and therefore does not mediate this auto-inhibition. The hinge regions of p85α and p85β also differ in length: In p85α, the region extends to 17 amino acids and in p85β to 18 amino acids (Figure 4A). In order to test for possible effects of these differences in sequence and length, we constructed cSH2 exchange mutants with the respective hinge region included (p85α/hβcSH2 and p85β/hαcSH2, Figure 4B). We also carried out one-amino acid deletions and substitutions in the respective hinge regions (p85α D615ins and p85β D612del, Figure 4F). Exchanging cSH2 domains plus hinge region had a significantly smaller effect on oncogenic activity compared to the exchange of the cSH2 domains alone (Figure 4C and 4D vs. Figure 3A, 3B and 3D). The length of the hinge region also appeared to have a mild effect in that adding an amino acid to the p85α hinge enhances oncogenic activity, and deleting an amino acid from the hinge region of p85β decreases focus formation (Figure 4F). We conclude that the strong functional differences of the p85α and p85β cSH2 domains can be significantly reduced by the hinge region between iSH2 and cSH2.

### Transforming activity is not correlated with protein stability

**Figure 5:**
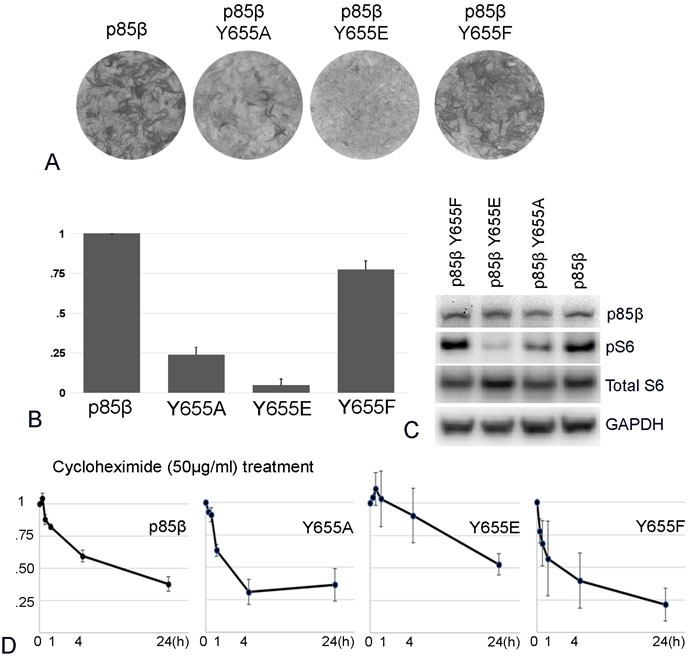
p85β protein stability and oncogenic activity **A.** Representative focus assays of p85β and p85β Y655 mutants. **B.** Efficiencies of cellular transforming activity of p85 Y655 mutants relative to the transforming activity of p85β. CEF were transfected RCAS(A) construct expressing p85β and the point mutations. Transforming activity was standardized to 0.5 μg transfected DNA. **C.** PI3K signaling in cells expressing p85β and p85β Y655 mutants as reflected by Western blots of S6 phosphorylated at Ser235/236. The data in Figure 5C are from a representative experiment that was repeated three times with consistent results (for details, see the Materials and Methods section). **D.** Time course of degradation of p85β and its Y655 mutant proteins as determined by cycloheximide (50 μg/ml) treatment. Protein concentrations were determined by Western blotting.

**Figure 6:**
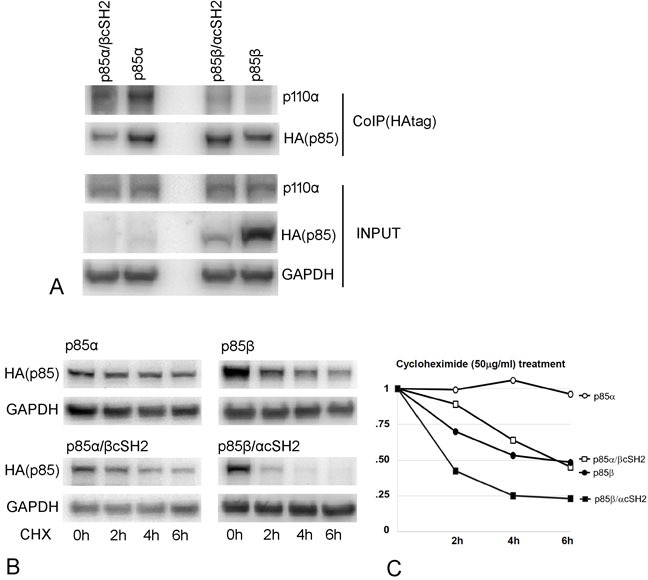
Protein interactions and stability of p85 cSH2 exchange mutants **A.** Co-immunoprecipitation of p110α with HA-tagged p85β, p85β/αcSH2, p85α and p85α/βcSH2. p110α interacts with wild type p85α, p85β and the cSH2 exchange mutants. **B.** Cycloheximide (50 μg/ml) treatment of p85α, p85β and the cSH2 exchange mutants. The Western blot indicates the protein expression level during cycloheximide (50 μg/ml) treatment compared to GAPDH as a control. **C.** Time course of p85 and exchange mutant degradation as determined by cycloheximide treatment.

The levels of free p85β in the cell are controlled by the F-box protein FBXL2 and the tyrosine protein phosphatase PTPL1 [15]. FBXL2 is a component of the SCF ubiquitin ligase complex. It binds to p85β, mediating substrate recognition of SCF, ubiquitination and proteasomal degradation. Whereas most SCF targets require phosphorylation for recognition, in the case of p85β, phosphorylation at Y655 inhibits the interaction with FBXL2 and increases stability. Dephosphorylation by PTPL1 enhances FBXL2 binding and degradation of p85β. In order to explore a potential relationship between protein stability and oncogenic activity in p85β wild type and exchange mutants, we mutated Y655 of p85β and determined the protein stabilities of these mutants and their oncogenic activities (Figures 5 and 6). The Y655A mutant, lacking the protection provided by phosphorylation, is less stable than wild type p85β, in accord with previously published data [15]. The Y655E mutation has a stabilizing effect, apparently inhibiting FBXL2 interaction more than the phosphorylatable tyrosine (Figure 5C and 5D). The unstable Y655A mutant surprisingly retains some transforming activity in cell culture as well as signaling activity as seen by phosphorylation of AKT (Figure 5A and 5B). In contrast, the stable Y655E mutant has lost virtually all transforming and signaling activities (Figure 5A and 5B). We then extended the protein stability studies to the cSH2 exchange constructs, first verifying that these constructs still interact with p110 (Figure 6A). The wild type p85α protein is more stable than p85β, possibly reflecting the FBXL2 interaction that is specific to the latter. The p85β/αcSH2 construct is the least stable, whereas the p85α/βcSH2 construct shows about the same stability as the p85β wild type protein (Figure 6B and 6C). Again, there is no correlation with oncogenic activity (compare Figure 6B and 6C with Figure 3B - 3D). The most stable protein, wild type p85α, lacks oncogenic activity. The two proteins with intermediate stability, wild type p85β and p85α/βcSH2, have the highest oncogenic activity of the four, and p85β/αcSH2 is unstable and lacks oncogenic activity. We conclude that there is no demonstrable correlation between protein stability and oncogenic activity.

### The iSH2 domains of p85α and p85β enhance PI3K oncogenic signaling

**Figure 7:**
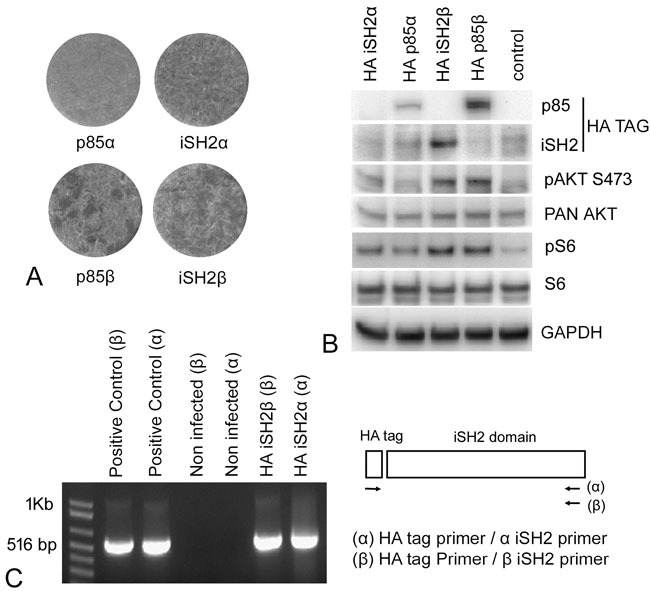
Expression of the isolated iSH2 domains enhances oncogenic and signaling activity of PI3K **A.** Representative focus assays of p85α, p85β, iSH2α and iSH2β. Both iSH2 domains induce a thickening of the cell sheet that is indicative of oncogenic transformation. The effect is not as pronounced as that induced by p85β, but is evident in comparison with the non-transforming p85α. **B.** Western blots documenting enhanced PI3K signaling with pAKT S473 and pS6. Expression of the iSH2α domain with the HA tag was not clearly demonstrable in standard Western blots and was therefore documented with qRTPCR **C.** The positive controls in this figure represent standard PCR from the DNA constructs; the HA iSH2α and iSH2β bands were generated by qRTPCR.

We cloned the p85α iSH2 (iSH2α) and p85β iSH2 (iSH2β) domains with an N-terminal HA-tag into the RCAS(A) vector and expressed these constructs in CEF. The expression of the iSH2 domains could not be detected by standard Western blotting using the HA-tag (Figure 7B), but was demonstrable using excessively long exposures that resulted in high background staining. We therefore verified iSH2 expression by quantitative RT-PCR (Figure 7C). Expression of the iSH2 domains in CEF led to oncogenic transformation. However, compared to the activity of p85β, the morphologic changes indicative of cellular transformation were weaker with the iSH2 domains, notably with iSH2α (Figure 7A). The level of PI3K signaling associated with the expression of iSH2α and iSH2β also showed enhancement as judged by phosphorylation of AKT at S473 and ribosomal protein S6 at the canonical C-terminal serine cluster (Figure 7B). Since it is known that binding of the iSH2 domains stabilizes p110 [16, 17], we examined p110 for an effect of iSH2 overexpression on protein stability using the cycloheximide chase described in Figure 6. No clear increase of p110 stability was found as a result of expressing either iSH2α or iSH2β.

## DISCUSSION

The classic view of p85 functions is that it has two roles in the PI3K complex. Binding of p85 to the ABD domain stabilizes p110, and binding of the nSH2 domain inhibits the catalytic activity of p110. *In vitro* biochemical studies have supported these two functions extensively as have studies of mutations and truncations of PIK3R1 [1, 3, 5–9, 12, 13, 16–22].

In this study, we analyzed truncations, deletions and domain hybrids of p85α and p85β for oncogenicity and signaling activity, documenting novel functional differences between the two isoforms. Our data show that all of the domains of p85α and p85β have functional consequences on PI3K activity and oncogenic potential in the context of whole cells.

We find that the SH3 domain exerts an inhibitory effect on oncogenic potential and PI 3-kinase signaling. In the case of p85α, this effect could be explained by the role of the SH3 domain in p85α homodimerization which in turn is required for the interaction between p85α and PTEN [10, 11, 23]. The same explanation could also apply to p85β, although binding of this isoform to PTEN has not yet been demonstrated. Deletion of the combined SH3 and RhoGAP domains resulted in loss of oncogenic activity. The deletions at the N-terminus would also affect homodimerization of p85α and possibly heterodimerization of p85α and p85β [11, 24]. Interestingly, a point mutation in the RhoGAP domain has previously been shown to activate the oncogenic potential of p85α [25]. The inactivity of the combined SH3-RhoGAP domain deletion is unexpected and may reflect some as yet unknown role of the RhoGAP domain in PI3K activity.

At the C-terminus of p85, the truncation of the cSH2 domain of p85α and p85β revealed an unexpected difference between the two isoforms. The cSH2 domain of p85α appears to have an inhibitory effect on oncogenic potency and signaling, whereas the cSH2 domain of p85β functions in the opposite direction: it is required for optimal transforming activity and functions as a stimulator of signaling. These opposing activities of the cSH2 domains are confirmed by domain exchange experiments: p85α with the cSH2 domain of p85β becomes oncogenic; in contrast, p85β with the cSH2 domain of p85α loses oncogenicity. These results are in contrast to the findings of Zhang et al. [13] who analyzed the structure of the p110β-p85β complex and identified inhibitory contacts of the p85β cSH2 domain and p110β. Zhang et al. confirmed this inhibition functionally by mutating the critical contacts and observing activation of PI3K. At this time, we have no explanation for this discrepancy. The possibility that truncation of the different cSH2 domains affects the conformation of the residual molecules differently is unlikely in view of the data from the cSH2 domain exchanges. These suggest that the positive and negative effects of the cSH2 domains reflect intrinsic properties of the domains. Speculative explanations for the discordance of results include the suggestion that deletion of the cSH2 domain in p85β could induce homodimerization which would take the protein out of the PI3K signaling pathway and result in decreased oncogenic and signaling activity. The activity of the p85α/βcSH2 construct could be explained by the known preference of p85α for p110α where the inhibitory contacts would not exist [22]. The cSH2 domain has also been highlighted by recent genetic studies. Mutations in this domain were identified in patients with insulin resistance uncoupled from dyslipidemia [26, 27]. The PI3K-activating function of the p85β cSH2 domain could play a significant role in determining the cancer phenotype, notably in those cancers where p85β is overexpressed or amplified. A special significance of the p85β cSH2 domain is also suggested by the fact that p85β is only infrequently truncated in cancer, whereas such truncations are common in p85α [28–30].

Larger truncations of C-terminal domains greatly attenuate the effects of the single cSH2 domain deletions, resulting in constructs of low activity for p85α as well as p85β. Interacting with p110, the nSH2 domain binds to the helical domain and the iSH2 domain associates with the adapter-binding and C2 domains of p110 to stabilize and inhibit the catalytic subunit [13, 16, 18, 20, 21]. Both SH2 domains play distinct roles in the regulation of PI3K activity [17]. By binding to pYXXM motifs in upstream activating proteins, principally receptor tyrosine kinases, nSH2 domains facilitate a change in p110 to the active conformation [6, 17, 19]. The cSH2 domain of p85α can also interact with IKK and PKC, resulting in phosphorylation of two serines in that domain with an inhibitory effect on PI3K signaling [31, 32]. Deletions that include the iSH2 domain would also abolish the interaction of p85 with BRD7 [33], interfering with a negative regulatory pathway of PI3K. The attenuating effect of the hinge regions in the cSH2 exchanges could be explained by suggesting that heterologous hinge regions might not position the cSH2 domains correctly on the recipient molecule. The activating activities observed with iSH2α and iSH2β could result from a stabilization of p110 or from reducing the interaction with wild type p85 and hence diminishing p85-mediated inhibition. We prefer the latter explanation, because we have not detected significant stabilization of p110 in iSH2-expressing CEF.

The observation that oncogenic and signaling activities are not affected by protein stability is counterintuitive. It could reflect a low minimal concentration threshold for an oncogenic protein to score in the cell system used. That requirement could be satisfied by the high rate of protein synthesis directed by the RCAS vector. Thus, the differences in protein stability would not affect transformation or signaling.

In summary, in this study we document important and distinct properties of the p85α and p85β isoforms. We confirm and extend the inhibitory function of the SH3 domain for PI3K-mediated transformation and signaling. We discover contrasting properties of the cSH2 domains and find no correlation between protein stability and oncogenicity as well as signaling activity in the investigated cell system.

## MATERIALS AND METHODS

### Cell culture and transfection

Chicken embryo fibroblasts (CEF) were prepared from single, pathogen-free White Leghorn embryos (Charles River, N. Franklin, CT) as previously described [34]. Transfection was performed with avian replication-competent retroviral vector RCAS(A) [35, 36] using the dimethyl sulfoxide/Polybrene method [34, 37, 38]. After two passages in the presence of serum, the cells expressing the recombinant RCAS viruses were harvested for further analysis.

### Oncogenic transformation

Transformation assays were carried out in six-well plates. CEF were transfected with DNA of the recombinant RCAS(A) constructs specified below using Lipofectamine 2000 (Thermo Fisher Scientific, Asheville, NC) and were kept in growth medium for 24 h before being overlaid with nutrient agar containing Ham's F-10 medium with 20% Earle's balanced salt solution, 0.6% SeaPlaque Agarose, 3% FBS, 1% heat-inactivated chicken serum, 9% tryptose phosphate broth, 1.8 mM glutamine, 89 U/mL penicillin, 89 μg/mL streptomycin, 1.1% DMSO. This mixture was applied every 2-3 days for 10 days, at which point the overlay was removed and the cell layer stained with crystal violet for quantitative evaluation of oncogenic activity by focus formation [39, 40]. In the figures, focus forming activities are standardized to 0.5 μg DNA and are expressed relative to the focus-forming activity of p85β. A summary of the raw data is presented in Supplementary Table S1.

### Constructs (See Supplementary Table S2)

cDNA of the human PIK3R1 and PIK3R2 genes was used to construct the expression vectors using the RCAS(A) backbone [36]. The C-terminal truncations of p85α and p85β and the p85β Y655 mutant constructs were generated by using the QuikChange site-directed mutagenesis kit (Agilent, Wilmington, DE). For the C-terminal truncations of p85β, amino acids G320, K435 and E600 were replaced with a stop codon (TAA); for the C-terminal truncations of p85α, amino acids K310, K438 and Y607 were replaced with a stop codon. For the Y655 mutants of p85β, Y655 was replaced with Y655A, Y655E or Y655F. Single amino acid insertions and deletions in the p85 hinge regions were generated by inserting Asp (D) at position 615 of p85α and deleting D612 in p85β. N-terminal truncation and domain exchange mutants were constructed by PCR or fusion PCR as described [41]. For the p85β N-terminal truncations, the forward primer was modified to add a translational start site (ATG) at residue positions 105 and 314. For the p85α N-terminal truncations, the forward primer was modified to add a translational start site (ATG) at positions 79 and 304. Domain exchange mutants used combinations of primers to generate DNA fragments using Platinum Pfx DNA Polymerase (Invitrogen, Carlsbad, CA) (Supplementary Table 2 and Supplementary Figure 1). The primer combinations were as follows: p85β/αcSH2 (primers A, B, C, D, see Supplementary Figure S1), p85β/αiSH2 (primers A, B, E, F, G, H), p85β/hαcSH2 (primers A, B, F, H), p85α/βcSH2 (primers I, J, K, L), p85α/βiSH2 (primers I, J, M, N, O, P), p85α/hβcSH2 (primers I, J, N, P). For the HA-tagged iSH2β and iSH2α DNA fragments were generated using Platinum Pfx DNA Polymerase (Invitrogen, Carlsbad, CA) (Supplementary Table S2). The mutated genes were subsequently cloned as SfiI DNA fragments into RCAS(A) [36]. The specified mutated p85 or wild type proteins were HA-tagged by PCR and cloned into the RCAS(A) vector. All clones were confirmed by sequencing and protein expression. N-terminal HA-tags do not affect protein function as confirmed by comparison with non-tagged protein in transformation assays.

### Statistical analysis

The experiments reported here were carried out with primary cells. For repeat experiments, cells routinely have to be derived from different avian embryos. Because starting conditions are then not identical, standard statistical analyses based on mean +/− standard deviation are not applicable. However, all these tests were repeated three times with consistent results, and the figures shown are from representative experiments.

### RT PCR

CEF were infected with the RCAS(A) vector expressing HA-iSH2α and HA-iSH2β at a MOI of 1; non-infected CEF cells were used as negative controls. After two passages in growth medium, total cellular RNA was isolated using TRIZOL (Invitrogen, Carlsbad, CA) according to the manufacturer's protocol. First strand cDNA was synthesized using a reverse transcription kit (SuperScript III Reverse Transcriptase, Invitrogen, Carlsbad, CA) with a random primer. PCR amplification was performed using Platinum Pfx DNA Polymerase (Invitrogen, Carlsbad, CA) with a HA-tag-specific primer and iSH2α and iSH2β C-terminal-specific sequence primers (Supplementary Table 2). The PCR products were identified by DNA electrophoresis. The positive controls were the RCAS(A) HA-iSH2α and HA-iSH2β vector DNA used as template in standard PCR to confirm product size.

### Western blots and antibodies

CEF were infected with RCAS(A)-expressing p85β, p85α, or the specified p85 mutants at a MOI of 1. After two passages in growth medium, cells were switched for overnight incubation to Ham's F-10 medium containing 0.5% FCS (calf serum) and 0.1% chicken serum, and this was followed by 2 h in basal F-10. At this point, protein samples were harvested. Western blotting was performed as described [2, 42], with minor modifications. Proteins were extracted from cells using ice-cold RIPA buffer (50 mM Tris·HCl pH 8, 100 mM NaCl, 0.5% Nonidet P-40, 0.5%, sodium deoxycholate, 0.1% SDS, 1 mM PMSF, 1 mM NaVO4, 1× Complete protease inhibitor mixture; Roche, Indianapolis, IN). Proteins were separated by 4-12% gradient SDS/PAGE (Invitrogen, Carlsbad, CA) using the MOPS buffer system. Separated proteins were transferred to PVDF membranes (Millipore, Billerica, MA) with transfer buffer and a transfer apparatus (Invitrogen, Carlsbad, CA). Transferred proteins were visualized with Ponceau S staining, blocked with 5% BSA in TBST. Primary antibodies were added as follows: p85β (MA1-21473, Thermo Fisher Scientific, Asheville, NC), p85α (#4257), HA-tag (#2367), AKT (#4685), AKT p-S473 (#4051), GAPDH (#2118) (Cell Signaling, Danvers, MA). Secondary antibodies were rabbit (No. 31462) or mouse (No. 31432) anti-HRP (Thermo Scientific, Asheville, NC). Western blots were visualized using HRP conjugates, and detection was performed using Super Signal West Pico Chemiluminescent Substrate (Thermo Fisher Scientific, Asheville, NC) according to the manufacturer's specifications.

### Protein stability

CEF were infected with RCAS(A)-expressing p85β, p85α, p85β Y655A, Y655E, Y655F, p85β/αcSH2 or p85α/βcSH2 at a MOI of 1. After two passages, the cells were treated with the translation inhibitor cycloheximide at 50 μg/ml to stop protein syntheses. Total protein samples were harvested using ice-cold RIPA buffer at 0, 0.25, 0.5, 1, 4 and 24 h after cycloheximide treatment for analyzing expression levels relative to GAPDH by Western blotting. We used the untreated sample as a standard. Relative protein stabilities were determined from Western blots and converted into line graphs. The error bars indicate standard deviations obtained in three independent experiments.

### Co-immunoprecipitation

CEF were infected with RCAS(A) virus that expresses HA-tagged p85β, p85α, p85β/αcSH2 or p85α/βcSH2 at a MOI of 1. After two passages, cells were rinsed with phosphate-buffered saline (PBS) and lysed in immunoprecipitation lysis buffer (20 mM Tris-Cl, pH 7.4, 150 mM NaCl, 0.5 mM EDTA, 0.5% NP-40, 1 mM NaVO4, 0.5 mM phenylmethlysulfonyl fluoride (PMSF) and 1× Complete protease inhibitor mixture; Roche, Indianapolis, IN). Cell lysates (0.5 to 1 mg of protein) were mixed with primary HA-tag antibody (#2367, Cell Signaling, Danvers, MA) and incubated overnight at 4°C with gentle agitation. The lysates were then incubated with protein A/G beads (sc-2003, Santa Cruz Biotechnology, Santa Cruz, CA) for 1 h at 4°C with agitation. The beads were washed three times with washing buffer (20 mM Tris pH 7.5,100 mM NaCl, 0.5% NP-40, 0.5 mM EDTA, 0.5 mM PMSF), bound proteins were eluted by boiling in 4XSDS sample (Invitrogen, Carlsbad, CA) and analyzed for p85-p110α interaction by Western blotting. The antibody against p110 used in the co-immunoprecipitation experiment was #4255 from Cell Signaling, Danvers, MA.

## SUPPLEMENTARY MATERIALS FIGURES AND TABLES






