# Supplementary material for: Domain analysis reveals striking functional differences between the regulatory subunits of phosphatidylinositol 3-kinase (PI3K), p85α and p85β
Source: Oncotarget. 2017 Aug 3;8(34):55863–76. doi: 10.18632/oncotarget.19866 (PMC5593529; doi:10.18632/oncotarget.19866)
Supplement: Supplementary file 1 [file oncotarget-08-55863-s001.pdf]

Domain analysis reveals striking functional differences between the regulatory subunits of phosphatidylinositol 3-kinase (PI3K), p85α and p85β

SUPPLEMENTARY MATERIALS

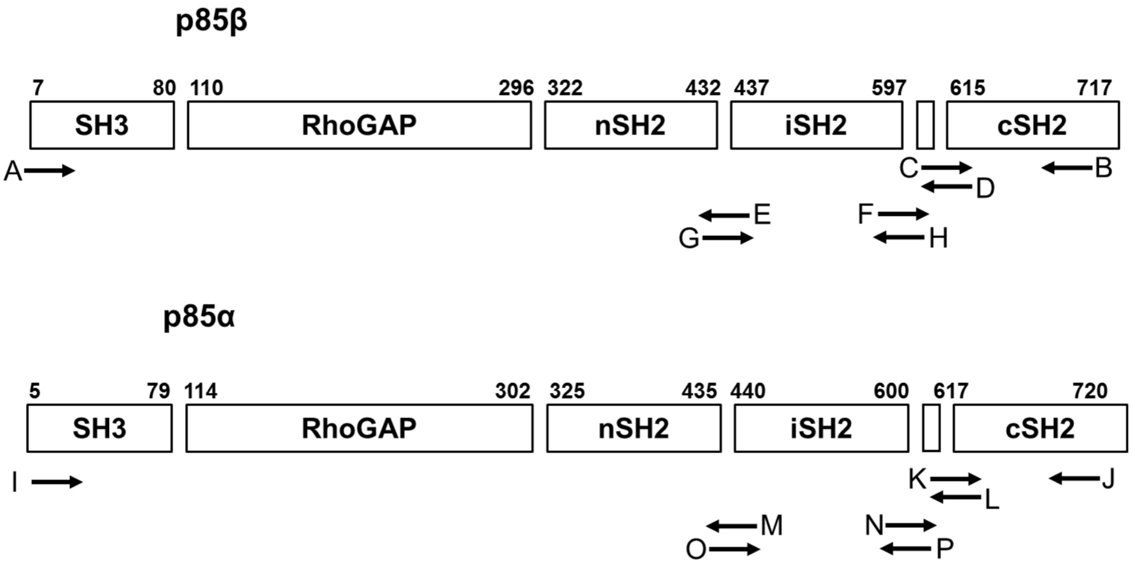

Supplementary Figure 1: Location of primers. The figure shows the positions of the primers listed in Table S2.

For Supplementary Tables see in Supplementary Files.
