# Supplementary material for: Domain analysis reveals striking functional differences between the regulatory subunits of phosphatidylinositol 3-kinase (PI3K), p85α and p85β
Source: Oncotarget. 2017 Aug 3;8(34):55863–76. doi: 10.18632/oncotarget.19866 (PMC5593529; doi:10.18632/oncotarget.19866)
Supplement: Supplementary file 2 [file oncotarget-08-55863-s002.docx]

Table S1: Oncogenic transformation by RCAS(A) constructs

N-terminal truncations:

|  | Av. focus count/5µg DNA |
| --- | --- |
| p85β | 30.0 |
| p85β ΔSH3 | 34.3 |
| p85β ΔSH3 Rho GAP | 3.3 |
| p85α | 0.6 |
| p85α ΔSH3 | 30.6 |
| p85α ΔSH3 Rho GAP | 3.0 |

C-terminal truncations:

| p85β | 30.6 |
| --- | --- |
| p85β ΔcSH2 | 2.6 |
| p85β ΔicSH2 | 3.0 |
| p85β ΔnicSH2 | 7.0 |
| p85α | 1.0 |
| p85α ΔcSH2 | 18.6 |
| p85α ΔicSH2 | 3.3 |
| p85α ΔnicSH2 | 2.3 |

SH2 exchange mutants:

| p85β | 32.0 |
| --- | --- |
| p85β/αiSH2 | 11.3 |
| p85β/αcSH2 | 3.0 |
| p85α | 1.6 |
| p85α/βiSH2 | 8.0 |
| p85α/βcSH2 | 15.3 |

cSH2 exchange mutants with hinge

| p85β | 33.3 |
| --- | --- |
| p85β/hαcSH2 | 14,3 |
| p85α | 1.6 |
| p85α/hβcSH2 | 2.0 |

Amino acid deletion or insertion mutants:

| p85β | 31.1 |
| --- | --- |
| p85β D612del | 9.3 |
| p85α | 1.6 |
| p85α D651ins | 17.6 |

p85β Y655 mutants:

| p85β | 35.5 |
| --- | --- |
| p85β Y655A | 8.3 |
| p85β Y655E | 1.6 |
| p85β Y655F | 27.3 |
