# Supplementary material for: Domain analysis reveals striking functional differences between the regulatory subunits of phosphatidylinositol 3-kinase (PI3K), p85α and p85β
Source: Oncotarget. 2017 Aug 3;8(34):55863–76. doi: 10.18632/oncotarget.19866 (PMC5593529; doi:10.18632/oncotarget.19866)
Supplement: Supplementary file 3 [file oncotarget-08-55863-s003.docx]

Table S2: Primers used in the generation of mutant constructs.

**Y655 point mutations and hinge region mutations:**

p85β Y655A Forward 5' CAGCCAGCGGGGCTGC**GCA**GCCTGCTCCGTGGTAG 3'

Reverse 5' CTACCACGGAGCAGGC**TGC**GCAGCCCCGCTGGCTG 3'

Y655E Forward 5' CAGCCAGCGGGGCTGC**GAG**GCCTGCTCCGTGGTAG 3'

Reverse 5' CTACCACGGAGCAGGC**CTC**GCAGCCCCGCTGGCTG 3'

Y655F Forward 5' CAGCCAGCGGGGCTGC**TTC**GCCTGCTCCGTGGTAG 3'

Reverse 5' CTACCACGGAGCAGGC**GAA**GCAGCCCCGCTGGCTG 3'

D612del Forward 5' GAGGACGAGGAC*****CTCCCGCACCACG 3'

Reverse 5' CGTGGTGCGGGAG*****GTCCTCGTCCTC 3'

D615ins Forward 5' GAAGATGATGAAGAT**GAC**TTGCCCCATCATGATG 3'

Reverse 5' CATCATGATGGGGCAA**GTC**ATCTTCATCATCTTC 3'

**C-terminal truncations:**

p85β G320* Forward 5' CACAGTCCTGGCCAATGGA**TAA**AGCCCACCCTCCCTGCAG 3'

Reverse 5' CTGCAGGGAGGGTGGGCT**TTA**TCCATTGGCCAGGACTGTG 3'

K435* Forward 5' GGACCAGATTGTC**TAG**GAGGACAGCG 3'

Reverse 5' CGCTGTCCTC**CTA**GACAATCTGGTCC 3'

E600* Forward 5' GCTGGGGATTAAAAAT**TAG**ACTGAGGACCAGTAC 3'

Reverse 5' GTACTGGTCCTCAGT**CTA**ATTTTTAATCCCCAGC 3'

p85α K310* Forward 5' CAGCACTGCCTCCT**TAA**CCACCAAAACC 3'

Reverse 5' GGTTTTGGTGGT**TAA**GGAGGCAGTGCTG 3'

K438* Forward 5' CCAACAGGATCAAGTTGTC**TAA**GAAGATAATATTGAAGC 3'

Reverse 5' GCTTCAATATTATCTTC**TTA**GACAACTTGATCCTGTTGG 3'

Y607* Forward 5' CACTGAAGACCAA**TAA**TCACTGGTGGAAGATG 3'

Reverse 5' CATCTTCCACCAGTGA**TTA**TTGGTCTTCAGTG 3'

p85β 105 ATG Forward 5’ AGGCCATTACGGCC**ATG**CCTGAGCCAGGCCTCACACTCCCCGA 3’

Reverse 5' AGGCCGAGGCGGCCAGAAATGAGGACCCCTGGATGT 3'

314 ATG Forward 5’ AGGCCATTACGGCC**ATG**ACAGTCCTGGCCAATGGAGGGAGC3’

Reverse 5' AGGCCGAGGCGGCCAGAAATGAGGACCCCTGGATGT 3'

p85α 79 ATG Forward 5’ AGGCCATTACGGCC**ATG**AGGAAAAAAATCTCGCCTCCCACA 3’

Reverse 5' AGGCCGAGGCGGCCATCATCGCCTCTGCTGTGCATATA 3'

304 ATG Forward 5’ AGGCCATTACGGCC**ATG**gcaccagcactgcctcctaaacca 3’

Reverse 5' AGGCCGAGGCGGCCATCATCGCCTCTGCTGTGCATATA 3'

**p85 wild type and domain exchange mutations (see Fig. S1):**

p85β Primer A 5’ AGGCCATTACGGCCATGGCGGGCCCTGAGGGCTTCCAGTACCGC 3’

Primer B 5' AGGCCGAGGCGGCCAGAAATGAGGACCCCTGGATGT 3'

p85β/αcSH2 Primer C 5’ ***ACTCATGGAGGACGAGGACGATCTCCCGCAT***CATGATGAGAAGACATGGAATGTTGGA 3’

Primer D 5’ TCCAACATTCCATGTCTTCTCATCATGAT***GCGGGAGATCGTCCTCGTCCTCCATGAGT*** 3’

p85β/αiSH2 Primer E 5’ ***AATTTTTTCCCTACAGCTTCAATATT***CTCCTTGACAATCTGGTCCTGCT 3’

Primer F 5’ ***AACGAGTGGTTGGGCAAT***GAAAAAAATGAGACTGAGGACCAGTA 3’

Primer G 5’ AGCAGGACCAGATTGTCAAGGAG***AATATTGAAGCTGTAGGGAAAAAATT*** 3’

Primer H 5’ TACTGGTCCTCAGTCTCATTTTT***TTCATTGCCCAACCACTCGTT*** 3’

HA-tag p85β Primer HA β 5’ AGGCCATTACGGCCATG*TACCCATACGATGTTCCAGATTACGCT*GCGGGCCCTGAGGGCTTCCAGTACCGC 3’

p85α Primer I 5' AGGCCATTACGGCC***ATGAGTGCTGAGGGGTACCAGTAC*** 3'

Primer J 5' AGGCCGAGGCGGCC***ATCATCGCCTCTGCTGTGCATATA*** 3'

p85α/βcSH2 Primer K 5’ TGGTGGAAGATGATGAAGATTTGCCC***CACCACGAGGAACGCACTTGGTACGTGGGCA*** 3’

Primer L 5’ ***TGCCCACGTACCAAGTGCGTTCCTCGTGGTG***GGGCAAATCTTCATCATCTTCCACCA 3’

p85α/βiSH2 Primer M 5, GCCCACTGCCTCCACGCTGTC***ATCTTCTTTGACAACTTGATCCTGT*** 3’

Primer N 5’ AAAATCAACGAGTGGCTGGGGATT***AACACTGAAGACCAATATTCACTG*** 3’

Primer O 5’ ***ACAGGATCAAGTTGTCAAAGAAGAT***GACAGCGTGGAGGCAGTGGGC 3’

Primer P 5’ ***CAGTGAATATTGGTCTTCAGTGTT***AATCCCCAGCCACTCGTTGATTTT 3,

HA-tag p85α primer HA α 5’ AGGCCATTACGGCCATG*TACCCATACGATGTTCCAGATTACGCT****AGTGCTGAGGGGTACCAGTAC*** 3’

**iSH2 domain constructs:**

iSH2β Forward 5’ AGGCCATTACGGCCATG*TACCCATACGATGTTCCAGATTACGCT*GACAGCGTGGAGGCAGTGGGCGCCCAGCTTAA 3’

Reverse 5’ AGGCCGAGGCGGCCTCAAATCCCCAGCCACTCGTTGATTTTCTTCTG 3’

iSH2α Forward 5’ AGGCCATTACGGCCATG*TACCCATACGATGTTCCAGATTACGCT*AATATTGAAGCTGTAGGGAAAAAATTACATG 3’

Reverse 5’ AGGCCGAGGCGGCCTCATTCATTGCCCAACCACTCGTTCAACTTCTT 3’

**RT-PCR primers:**

HA tag Primer 5’ ATGTACCCATACGATGTTCCAGATTACGCT 3’

α iSH2 Primer 5’ TCATTCATTGCCCAACCACTCGTTCAACTTCTT 3’

β iSH2 Primer 5, TCAAATCCCCAGCCACTCGTTGATTTTCTTCTG 3’

Bold: Mutation and addition

Underline: SfiI site (restriction site)

Bold/Italic: p85α

Underline/Italic: HA-tag

*****: Deletion
